# Genital herpes evaluation by quantitative TaqMan PCR: correlating single detection and quantity of HSV-2 DNA in cervicovaginal lavage fluids with cross-sectional and longitudinal clinical data

**DOI:** 10.1186/1743-422X-7-328

**Published:** 2010-11-18

**Authors:** Bulbulgul Aumakhan, Andrew Hardick, Thomas C Quinn, Oliver Laeyendecker, Stephen J Gange, Chris Beyrer, Christopher Cox, Kathryn Anastos, Mardge Cohen, Ruth M Greenblatt, Daniel J Merenstein, Howard Minkoff, Marek Nowicki, Charlotte A Gaydos

**Affiliations:** 1 Johns Hopkins Bloomberg School of Public Health, Baltimore, MD, USA; 2 Johns Hopkins University School of Medicine, Baltimore, MD, USA; 3 Laboratory of Immunoregulation, National Institute of Allergy and Infectious Diseases, National Institutes of Health, Bethesda, MD, USA; 4 Albert Einstein College of Medicine, Bronx, NY, USA; 5 Cook County Medical Center, Chicago, IL, USA; 6 University of California, San Francisco School of Medicine, San Francisco, CA, USA; 7 Georgetown University Medical Center, Washington, D.C., USA; 8 Maimonides Medical Center and SUNY Downstate, Brooklyn, NY, USA; 9 University of Southern California, Los Angeles, CA, USA

## Abstract

**Objective:**

To evaluate the utility of a single quantitative PCR (qPCR) measurement of HSV (HSV-1&2) DNA in cervicovaginal lavage (CVL) specimens collected from women with predominantly chronic HSV-2 infection in assessing genital HSV shedding and the clinical course of genital herpes (GH) within a cohort with semiannual schedule of follow up and collection of specimens.

**Methods:**

Two previously described methods used for detection of HSV DNA in mucocutaneous swab samples were adapted for quantification of HSV DNA in CVLs. Single CVL specimens from 509 women were tested. Presence and quantity of CVL HSV DNA were explored in relation to observed cross-sectional and longitudinal clinical data.

**Results:**

The PCR assay was sensitive and reproducible with a limit of quantification of ~50 copies per milliliter of CVL. Overall, 7% of the samples were positive for HSV-2 DNA with median log_10 _HSV-2 DNA copy number of 3.9 (IQR: 2.6-5.7). No HSV-1 was detected. Presence and quantity of HSV-2 DNA in CVL directly correlated with the clinical signs and symptoms of presence of active symptomatic disease with frequent recurrences.

**Conclusion:**

Single qPCR measurement of HSV DNA in CVL fluids of women with chronic HSV-2 infection provided useful information for assessing GH in the setting of infrequent sampling of specimens. Observed positive correlation of the presence and quantity of HSV-2 DNA with the presence of active and more severe course of HSV-2 infection may have clinical significance in the evaluation and management of HSV-2 infected patients.

## Introduction

Genital herpes (GH) is a common chronic sexually transmitted infection worldwide with substantial morbidity [1,2] caused mainly by Herpes Simplex Virus Type 2 (HSV-2) and sometimes by HSV-1. Women, in particular, are disproportionately affected. GH is also commonly found among Human Immunodeficiency Virus (HIV) infected individuals in whom it is associated with increased HIV replication [3,4].

The majority of HSV-2 infected individuals is 'asymptomatic' or unaware of infection [5,6]. Those with symptomatic HSV-2 can experience recurrent episodes of genital lesions that appear to diminish in severity and frequency over time [7-9]. Most individuals with chronic HSV-2 have mild or asymptomatic infection.

Cell culture isolation of HSV is the preferred diagnostic test, usually used in conjunction with symptomatic primary or first clinical episode. However, its sensitivity for recurrent or healing lesions is low. More recently, PCR- based methods have been actively investigated for the detection of HSV DNA in mucocutaneous lesions and have shown to be superior to viral culture [10-12]. PCR has also been shown to be more sensitive in detecting asymptomatic shedding or shedding episodes in the absence of clinically obvious lesions [13-16]. Nevertheless, the potential utility of broad based application of PCR based techniques in the evaluation and management of HSV-2 infected patients, especially of those with longstanding and/or asymptomatic GH, is less clear given the plausibility of reduced genital shedding over time. In addition, the essential goal of most PCR assays was detection, i.e. determining the presence or absence of HSV target nucleic acid sequences in the sample. However, for pathogenesis studies and clinical management purposes, including prognosis or determining optimal drug regimens, quantification of actual viral load may be useful. Data on the usefulness of quantification of HSV DNA in genital secretions, perhaps due to mild nature of most HSV-2 infections, is limited and restricted mainly to evaluating clinical and virologic efficacy of antiviral compounds and defining the threshold of HSV infectivity as a potential factor in the transmissibility of infection [17-22]. Nevertheless, available evidence suggests that HSV-2 viral titer in genital secretions can be a useful means for disease monitoring purposes. A study by Filen et al., for example, found that first episodes of GH were associated with significantly higher viral loads compared to recurrent or atypical cases [9]. Yet, other studies doubt the usefulness of monitoring HSV loads in clinical samples [21,23]. Some of the challenges in ascertaining these issues are related to intermittent nature and wide variability in the frequency and amount of HSV shedding observed among infected individuals. Many investigators use repeated and frequent sampling up to multiple times a day to overcome these challenges [24]. However, for practical reasons, not all research and clinical settings can easily implement such an approach and, hence, the clinical usefulness of quantitative PCR (qPCR) methods, especially for those with established chronic GH and in the setting of infrequent sampling of specimens, is unclear.

Therefore, using quantitative PCR technique, we aimed to explore the usefulness of assessing genital HSV infection by single qPCR measurement of HSV DNA in cervicovaginal lavage (CVL) specimens of women with mostly longstanding HSV-2 infection within the setting of a research cohort with semiannual scheduling of follow up and specimen sampling. The presence and quantity of CVL HSV DNA were explored in relation to observed cross-sectional and longitudinal clinical data.

## Methods

### Study population and specimens

The study population consisted of HIV infected and uninfected participants of Women's Interagency HIV Study (WIHS), a multicenter cohort study of HIV in women across six sites in the US (Los Angeles, CA; Washington, DC; San Francisco, CA; New York City/Bronx, NY; Brooklyn, NY; and Chicago, IL). WIHS enrolled 2059 HIV infected and 569 high risk HIV uninfected women between October 1994 and November 1995 [25]. At enrollment, over 90% of WIHS participants were seropositive for HSV-1 and more than 80% of HIV infected women seropositive for HSV-2. HSV serostatus was determined by HSV type specific antibodies by glycoprotein G-based enzyme immunoassay (gG-EIA, Gull Laboratories, Salt Lake City, Utah). Negative and equivocal results were confirmed by Western Blot [26]. Gynecological examination included assessment for genital tract infections and genital tract dysplasia as previously described [27]. Self-reports of GH sores and observations of presence of lesions, sites of the lesions and whether the lesions were observed at multiple (three or more) locations were collected at each study visit. CVL specimens were collected by flushing the cervix with 10 ml sterile normal saline aspirated from the posterior vaginal fornix. The specimens were then transported to the processing laboratory on ice within 24-26 hours and 1 ml aliquots were stored at a central repository at -70°C. Whole unspun and unfractionated CVL was used for this study.

Total of 509 single CVL samples from 509 women were retrieved from repository for testing. Ten samples each from dual positive (HIV+/HSV+), HIV only (HIV+/HSV-), HSV only (HIV-/HSV+) positive women and 40 samples from dual negative (HIV-/HSV-) women were retrieved from the baseline visit to use in the assay validation. The rest were selected based on the following criteria: 1) had known baseline HSV serology status; 2) had information on self reported history of GH sores, physical and gynecological exams; 3) had at least one follow-up visit since the baseline; and 4) had sufficient volume of more than 5 ml CVL available to preserve the specimens. To assess the correlation of the initial or 'baseline' CVL HSV DNA titer with the number of subsequent lesion recurrences, we identified eligible samples from women who had multiple (> 1) recurrent episodes of lesion outbreaks (referred thereafter as lesion-episode) observed during the follow up. For these women, CVL sample from the earliest available lesion-episode was retrieved for testing and considered as a 'baseline' episode. Since the earliest available lesion-episode is different for each woman, the visits from which samples were pulled ranged from 1 to 24 with the median visit number of 3 (IQR: 1-8).

### Extraction of HSV DNA

CVL fluids were thawed at room temperature. DNA was extracted by QIAamp DNA blood minikit from 200 μl of whole CVL (Qiagen, Valencia, California) using the Blood and Body Fluid Spin Protocol. The DNA was eluted into 55 μl of Qiagen AE buffer. Each extraction included positive control HSV isolates (HSV-1 strain GHSV-UL46 and HSV-2 strain MS, ATCC, Manassas, VA) and RNase- and DNase-free water as the negative control.

### Preparation of HSV DNA standards

Ten-fold serial dilutions were prepared with commercial HSV-1&2 quantified DNA (ABI Advanced Technologies, Inc., Columbia, Maryland) to generate a standard curve. The DNA stocks were serially diluted with RNase- and DNase- free water and/or with CVL fluids pooled from HIV (+) and HIV (-) women whose CVLs were negative for HSV-1&2 DNA. To avoid repeated freeze-thaw of the DNA stock which could negatively affect the reproducibility of the assay, single use panels of serial dilutions were prepared immediately upon receipt of the DNA stock and stored at -20°C until further use. Standards were analyzed in duplicates and used to generate a standard curve as well as a positive control for each PCR run.

### Primers, probes and target sequence for amplification

Primers were adapted from two different sources. The forward primer (GbTypF: 5'-CGC ATC AAG ACC ACC TCC TC-3') was as described by Corey L. et al. [28]. The reverse primer (HSV1&2-R: 5'-AGC TTG CGG GCC TCG TT-3') and probes (HSV1-probe: 5'-CGG CCC AAC ATA TCG TTG ACA TGG C-3' and HSV2-probe: 5'-CGC CCC AGC ATG TCG TTC ACG T-3') were as described by Namvar et al. [29]. The probe region differs by 5 nucleotides and was previously shown to differentiate between HSV-1 and HSV-2 without cross-reactivity [29]. Probes were labeled at the 5'-end with FAM or VIC and at the 3'- end with TAMRA. Primers allowed amplification of a highly specific 155-nucleotide region of gB envelope gene homologous for HSV-1&2 which represented the summed extension of overlapping target sequences used by the two groups.

### TaqMan PCR

The final 50 μl PCR reaction mix contained 25 μl of 2× TaqMan universal master mix (PE Applied Biosystems, Foster City, CA), 900 nM of each primer, 100 nM of each probe and 10 μl of sample DNA. PCR was performed using an ABI 7900 HT sequence detection system (PE Applied Biosystems, Foster City, CA) with the following cycling conditions: incubation for 2 min at 50°C (uracil-*N*-glycosylase digestion) and denaturation at 95°C for 10 min followed by 45 cycles of 15 s denaturation at 95°C and 60 s annealing/extension at 58°C. Specimens were blinded to clinical information and run in duplicate. A sample was considered positive if the detected quantity was above or equal to assay limit of quantification in both replicates.

### Statistical analysis

Assay performance was evaluated using within and between assay measures of efficiency (slope of standard curve), linearity (R-square) and reproducibility (mean threshold (Ct) values, standard deviation (SD) and coefficient of variation (CV)) from standard curve data. Limit of detection (LoD) and limit of quantification (LoQ) were estimated using the delta method to approximate the relative precision of the estimated concentration as previously described [30]. Values of HSV-2 DNA were log_10 _transformed for analyses. Proportions with detectable HSV DNA by clinical markers of genital HSV infection were compared using chi-square and median quantities by Wilcoxon rank-sum tests. The markers were HSV-2 seropositivity, self report of GH lesions, the presence of any lesions and/or lesions clinically suspected as herpetic. To assess whether there is any relationship between the initial 'baseline' HSV-2 viral load and subsequent clinical course of GH, the correlation between the CVL HSV-2 DNA titer and the total number of lesion-recurrences observed during the subsequent follow up was explored. Duration of subsequent follow up was determined by the total number of follow up visits observed since the detection of HSV DNA in CVL. Ratio of frequency of subsequent lesion-episodes on duration of follow up was used to account for varying lengths of follow up among women. Pearson's r or Spearman's rho were used to estimate the correlations of interest. P-values of < 0.1 were considered significant. Statistical analyses were carried out using STATA 10.1 software (STATA Corporation, College Station, Texas). Graphs were created using GraphPad Prism Software, v. 5.03 (GraphPad Software, La Jolla, California).

## Results

### Assay performance

For HSV-1, the Ct values ranged from 21.62 for log_10_5 to 35.68 for log_10_1 with an average slope of -3.22 (range: -3.17 to -3.27). For HSV-2, the corresponding Ct values ranged from 23.74 to 38.50 with average slope of -3.33 (range: -3.23 to -3.49) indicating high efficiencies for both HSV types. The intra-assay CV (Ct) values for five dilutions of HSV-1&2 ranged from 0.02% to 4.26%. The inter-assay CV (Ct) ranged from 0.1% to 1.3%. R-square values for all runs were ≥ 0.99. Standards were stable with consistent Ct values for all concentrations in multiple runs performed over the course of 6 months. No significant differences were observed in Ct values between water and CVL diluted standards (≤ 1-2 Ct difference). HIV status did not influence the test performance. Data for HSV-2 are shown in Table 1.

**Table 1:** Assay Reproducibility (HSV-2)

				**Intra-assay**			**Inter-assay**		
					
**# copies per assay**	**Run**	**Ct**_**1**_	**Ct**_**2**_	**mean Ct**	**Ct SD**	**CV (Ct)**	**mean Ct**	**Ct SD**	**CV (Ct)**
	
150,000	1	24.11	23.99	24.05	0.08	0.34	24.05	0.11	0.46
	2	24.18	24.13	24.15	0.04	0.15			
	3	24.12	23.74	23.93	0.27	1.13			
									
15,000	1	27.34	27.28	27.31	0.04	0.14	27.25	0.16	0.57
	2	27.46	27.25	27.36	0.15	0.55			
	3	26.97	27.16	27.07	0.13	0.50			
									
1,500	1	30.67	30.56	30.62	0.08	0.25	30.76	0.14	0.45
	2	30.97	30.81	30.89	0.12	0.38			
	3	30.81	30.74	30.77	0.05	0.16			
									
150	1	34.82	33.90	34.36	0.66	1.91	34.30	0.25	0.74
	2	34.50	34.52	34.51	0.01	0.03			
	3	34.09	33.94	34.02	0.10	0.31			
									
15	1	37.09	37.44	37.27	0.25	0.67	37.41	0.15	0.41
	2	37.83	37.32	37.57	0.36	0.96			
	3	38.50	36.25	37.38	1.59	4.26			

### Limit of detection and limit of quantification

One to 1.5 copies per reaction were detected 50% of the time and 10 copies were detected in 100% of the runs. Thus, the LoD was considered as 1-2 copies/assay or 20-40 copies/ml. The LoQ for HSV-2 was ~2.3 copies per reaction corresponding to ~47 copies per mL of CVL and the LoQ for HSV-1 was ~6.4 copies per reaction or ~127 copies per mL of CVL. The higher LoQ observed for HSV-1 was due to slightly lower precision of the estimates in the linear regression compared to HSV-2. At least 6 replicates for each concentration from multiple runs were used to estimate LoQ.

### Study population

The study population consisted of 379 (74%) dually infected (HIV+/HSV+), 22 (4%) HIV only (HIV+/HSV-), 68 (13%) HSV only (HIV-/HSV+) and 40 (8%) neither HIV nor HSV (HIV-/HSV-) infected individuals (Table 2). Median baseline age of women was 35 years. HSV seropositive women were predominantly African American and significantly older as opposed to seronegative women. Intravenous drug use and heterosexual risk were the commonly identified routes of HIV exposure among HSV seropositive women. Median follow up of women was 24 visits (IQR: 14-24).

**Table 2:** Demographic and risk characteristics of the 509 women by HIV/HSV serostatus

Definition	HIV+/HSV+ N = 379 (74%)	HIV+/HSV- N = 22 (4%)	HIV-/HSV+ N= 68 (13%)	HIV-/HSV- N = 40 (8%)
Median age at baseline, years (IQR)	38 (33-42)	36 (28-40)	34 (28-40)	26(22-30)
				
Race n (%)				
African American	225 (59%)	6 (27%)	45 (66%)	13 (33%)
Hispanic	79 (21%)	3 (14%)	15 (22%)	7 (18%)
White	55 (15%)	11 (50%)	6 (9%)	18 (45%)
Other	20 (5%)	2 (9%)	2 (3%)	2 (5%)
				
Risk exposure				
Intravenous drug use	135 (36%)	6 (27%)	16 (24%)	8 (20%)
Heterosexual risk	149 (39%)	12 (55%)	11 (16%)	10 (25%)
Transfusion risk	10 (3%)	0 (0%)	1 (1%)	1 (3%)
No identified risk	85(22%)	4 (18%)	40 (59%)	21 (53%)
				
Lifetime # of sex partners				
0-1	16 (4%)	1 (5%)	3 (4%)	1 (3%)
2-4	51 (13%)	5 (23%)	13 (19%)	6 (15%)
5-9	81 (21%)	8 (36%)	19 (28%)	10 (25%)
10-50	122 (32%)	6 (27%)	19 (28%)	21 (53%)
> 50	108 (28%)	2 (9%)	14 (21%)	2 (5%)
missing	1 (0.3%)	--	--	--

### HSV-2 DNA detection by HIV/HSV status

Overall, 35 (7%) individuals were positive for genital HSV-2 DNA with a median log_10 _DNA copy number of 3.9 (IQR: 2.6 - 5.7). No HSV-1 was detected. Log transformed values of HSV-2 DNA were normally distributed (Shapiro-Wilk normality test p = 0.406). By HIV/HSV status, HIV+/HSV+ group had 27 women with detectable HSV-2 DNA (n = 379, 7%, median log_10 _HSV-2 DNA = 4.4, IQR: 2.6-5.9), HIV-/HSV+ group - 4 (n = 68, 6%, median log_10 _HSV-2 DNA = 2.8, IQR: 2.1-4.0) and HIV-/HSV- group - 4 women positive for HSV-2 DNA (n = 40, 10%, median log_10 _HSV-2 DNA = 3.7, IQR: 3.4-5.2). The number of HIV+/HSV- women was small (n = 22) and none had detectable HSV-2 DNA.

### HSV-2 DNA detection by clinical markers GH infection

Detection was highest for lesions clinically suspected as herpetic, 27% (p < 0.001) and 8% for presence of any lesions (Table 3). About 6% of those with lesions not identified as herpetic were positive for CVL HSV-2 DNA.

**Table 3:** CVL HSV-2 DNA detection by markers of genital herpes

Definition	Status **(+/-)**^**a**^	HSV-2 DNA (+), n/N (%)	*p-value*
HSV-2 serostatus	+	28/356 (8%)	0.251
	-	7/153 (5%)	
Self report of GH sores	+	4/42 (10%)	0.518
	-	31/467 (7%)	
Any lesions^b^	+	30/394 (8%)	0.296
	-	5/114 (4%)	
Herpetic lesions^c^	+	7/26 (27%)	0.001
	-	28/481 (6%)	

^a ^(+) condition present; (-) condition absent

^b ^any genital lesions vs. no any lesions

^c ^lesions clinically suspected as herpetic vs. no lesions or lesions not suspected as herpetic

### 'Baseline' CVL HSV-2 viral load and subsequent clinical course

**Figure 1:**
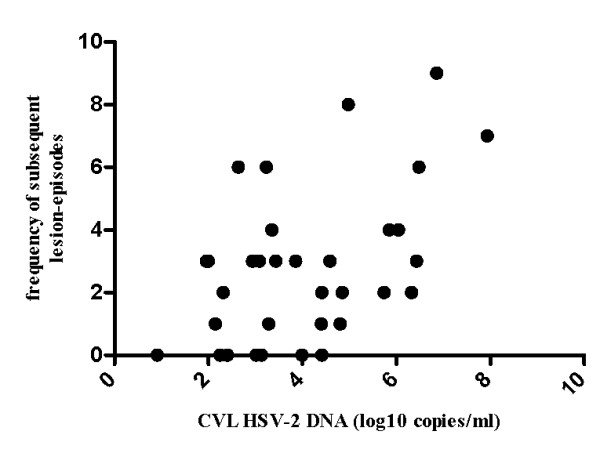
**Correlation between the frequency of subsequent lesion-episodes and CVL HSV-2 DNA titer, Pearson r = 0.48, p = 0.005**.

**Figure 2:**
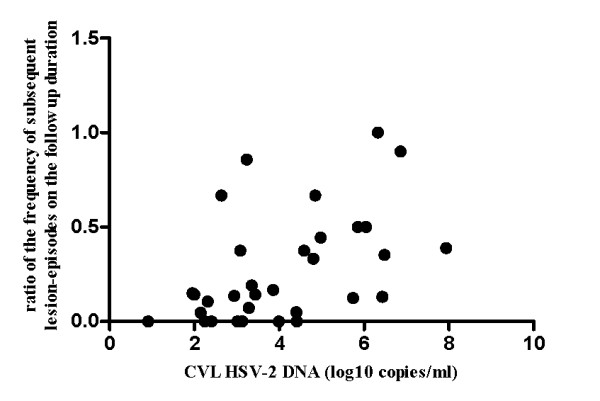
**Correlation between the ratio of the frequency of subsequent lesion-episodes on duration of follow up and CVL HSV-2 DNA titer, Pearson r = 0.50, p = 0.004**.

**Figure 3:**
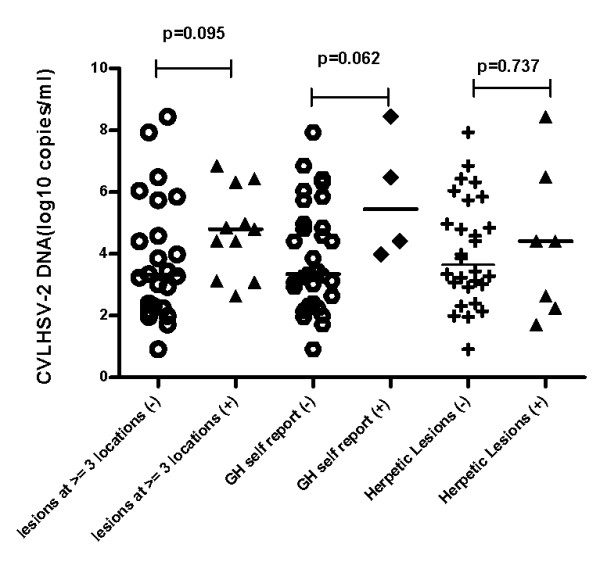
**Median CVL HSV-2 DNA titer by presence of lesions at 3 or more locations, presence of self reported history of genital herpes sores and presence of herpetic lesions**.

Positive correlation was observed between the CVL HSV-2 DNA load and the frequency of lesion-episodes observed during the subsequent follow up (Pearson r = 0.48, p = 0.005, Figure 1). Strength of the correlation did not change when the ratio of lesion-episodes frequency on the duration of follow up was used to account for varying lengths of follow up (Pearson r = 0.50, p = 0.004, Figure 2). There was also no correlation between the length of subsequent follow up and quantity of HSV-2 DNA (Spearman's rho = - 0.003, p = 0.852). Although there were no differences in the detection status, quantitatively, women with positive history of GH sores and lesions identified at three or more locations tended to have higher median quantities of HSV-2 DNA that were statistically significant at alpha level of 0.1 (Figure 3). Women with clinically suspected herpetic lesions had slightly higher median HSV-2 DNA titer but the difference did not reach statistical significance of p < 0.1. No quantitative differences by other markers were observed.

### HIV and HSV detection

Detailed analysis of the association between HIV and HSV detection including multivariate regression was described elsewhere (Aumakhan B, Gange SJ, Beyrer C, Gaydos CA, Minkoff H, Merenstein DJ, Cohen M, Anastos K, Greenblatt RM, Nowicki M, Quinn TC: Quantitative and qualitative correlates of cervicovaginal HSV-2 shedding among HIV infected women in Women's Interagency HIV Study, submitted). Briefly, trend for reduced detection of HSV-2 DNA with higher CD+ T cell counts was observed (p-value for trend = 0.08). No significant associations were observed with HIV viral load and use of antiretroviral therapy.

## Discussion

We explored the correlation of the presence and quantity of HSV-2 DNA in cervicovaginal fluids collected from women with predominantly established genital herpes infection with clinical manifestations observed at the visit (cross-sectionally) and over the course of follow up (longitudinally) using real time PCR technique. The PCR assay adapted two previously reported methods [28,29] used for detection and typing of HSV DNA in mucocutaneous swab samples to quantification of HSV DNA in CVL samples. The combination of primers and probes from two different sources was a result of preliminary review of primers and probes from reported methods during which it was determined that the target sequences of these two methods overlapped resulting in a final amplicon of 155-nucleotide region of glycoprotein B gene highly specific for HSV-1 and HSV-2 differentiation. Two type specific forward primers used by Namvar et al. [29] were conveniently replaced by one common type primer described by Corey et al. [28] and the assay was implemented using the absolute quantification guidelines recommended by the manufacturer (ABI 7900 HT SDS, PE Applied Biosystems, Foster City, CA).

Overall, we found a 7% HSV-2 DNA detection rate in the tested samples. Despite testing for HSV-1, no HSV-1 DNA was detected. Herpetic lesions had the most correlation with the probability of detectable HSV-2 DNA in CVL with 27% positivity rate. Although this finding may not be surprising, the main point of this observation is the extent of this correlation in this particular population and what to expect if broader categories, such as presence of any lesions, are used. The latter was associated with 8% positivity rate.

Four HIV-/HSV- women tested positive for HSV-2 DNA in CVL suggesting that they may have had primary genital HSV-2 infection. Three of them had multiple lesion-episodes observed during the subsequent follow up. However, only one had active lesion at the time of sampling and one reported positive history of genital sores in the past 6 months. The individual with the active lesion had the highest viral load with log10 HSV-2 DNA copy number of 6.4. Follow up measurement of serum anti-HSV-2 Ig G will be needed to confirm any subsequent seroconversion.

An interesting finding is the significant correlation observed between the 'baseline' CVL HSV-2 DNA load and the frequency of subsequent lesion recurrences observed during the follow up, which suggests that high HSV-2 load could be associated with frequent reactivations. Absence of the correlation between the length of subsequent follow up and HSV-2 DNA titer suggests that this association was not due to varying lengths of follow up. Trend towards higher median HSV-2 DNA titer with the presence of lesions at multiple locations could indicate that HSV-2 viral load plays a role in the severity of GH clinical expression. Although only 10% of women with self reported positive history of GH sores had detectable HSV-2 DNA, they tended to have higher HSV-2 DNA copy numbers compared to women without such history, which suggests that more readily recognizable lesions may harbor high levels of infectious virus. Additional studies with a larger number of positive end-points will be needed to validate these results. Nevertheless, findings of this study were the basis for classifying HSV-2 infected women into groups of gradient degree of GH clinical activity (determined by the presence/absence of active symptomatic disease with multiple recurrences) in another study by our group, in which we observed direct dose dependent association between classic markers of HIV disease progression (CD4+ T cell count, HIV RNA load) and a degree of HSV-2 clinical activity, which lends additional support to these results [31].

Our 7% of HSV-2 DNA detection rate in CVL may seem low compared to some other reports that measured genital HSV shedding using CVL specimens [32-34]. This may have been due to differences in the methods of CVL collection employed, sampling frequency or the population characteristics in which these assays were utilized. It is also lower than estimates of HSV shedding reported in previous WIHS study by Augenbraun et al. [35]. However, direct comparison between this and the previous study may not be cogent as studies used differed selection criteria for enrolling participants as well as different specimen types and sampling strategies.

Despite this limitation, the study has several unique strengths. First, although HSV-2 shedding was measured at single time point, we used rich longitudinal clinical data accumulated by WIHS over many years to link our PCR results with the observed clinical course of GH in these women. Second, as many studies explore HSV-2 using frequent sampling such as daily or even multiple sampling in a day [36,37], these results point to potential feasibility of studies of HSV-2 natural history in the settings with a less frequent sampling schedule and collection of data.

In summary, single qPCR measurement of HSV DNA in CVL specimens among women with chronic HSV-2 infection can provide useful information for assessing genital herpes in the setting of infrequent sampling of specimens. Observed positive correlation of the presence and quantity of HSV-2 DNA with active symptomatic disease with frequent reactivations suggests that HSV-2 quantification could be a useful tool in evaluating HSV-2 infected patients with chronic genital herpes and may guide better antiviral therapy.

## Competing interests

The authors declare that they have no competing interests.

## Authors' contributions

BA, TCQ, SJG, CB, CAG conceived and designed the study. BA, AH, OL, CAG performed the assay design and experiments. BA, SJG, CC carried out statistical analysis. KA, MC, RMG, DJM, HM, MN, SJG contributed samples and data. BA wrote initial draft of the manuscript. All authors read and approved the final manuscript.
